# Diagnostic Power of Vascular Endothelial Growth Factor and Macrophage Colony-Stimulating Factor in Breast Cancer Patients Based on ROC Analysis

**DOI:** 10.1155/2016/5962946

**Published:** 2016-07-03

**Authors:** Monika Zajkowska, Edyta Katarzyna Głażewska, Grażyna Ewa Będkowska, Przemysław Chorąży, Maciej Szmitkowski, Sławomir Ławicki

**Affiliations:** ^1^Department of Biochemical Diagnostics, Medical University of Bialystok, 15-089 Bialystok, Poland; ^2^Department of Esthetic Medicine, Medical University of Bialystok, 15-089 Bialystok, Poland; ^3^Department of Hematological Diagnostics, Medical University of Bialystok, 15-089 Bialystok, Poland; ^4^Department of Surgery, Public Health Hospital, 19-100 Monki, Poland

## Abstract

Breast cancer (BC) is the most common malignancy in women. Vascular endothelial growth factor (VEGF) has been described as an important regulator of angiogenesis which plays a vital role in the progression of tumor. Macrophage colony-stimulating factor (M-CSF) is a cytokine whose functions include regulation of hematopoietic lineages cells growth, proliferation, and differentiation. We investigated the diagnostic significance of these parameters in comparison to CA15-3 in BC patients and in relation to the control group (benign breast tumor and healthy women). Plasma levels of the tested parameters were determined by ELISA and CA15-3 was determined by CMIA. VEGF was shown to be comparable to CA15-3 values of sensitivity in BC group and, what is more important, higher values in early stages of BC. VEGF was also the only parameter which has statistically significant AUC in all stages of cancer. M-CSF has been shown to be comparable to CA15-3 and VEGF, specificity, and AUC values only in stages III and IV of BC. These results indicate the usefulness and high diagnostic power of VEGF in the detection of BC. Also, it occurred to be the best candidate for cancer diagnostics in stages I and II of BC and in the differentiation between BC and benign cases.

## 1. Introduction

Breast cancer (BC) is an important health problem worldwide. Each year the incidence rate of this disease increases significantly. In 2015, only in the United States about 231,840 women were diagnosed with BC and 40,290 of them died [1]. This disease may appear at any age, yet a particularly high risk is related to females after 50 years of age, what is correlated with menopausal hormonal changes [2].

The crucial factor influencing a successful treatment and survival rate of BC patients is early diagnosis. Biochemical detection of this tumor is nowadays based on markers such as CA 15-3, CEA, and CA 27.29 [3]. In view of their insufficient specificity and sensitivity at the initial type of BC, scientists around the world perform intensive research to find better biomarkers whose levels would correlate with the presence and stage of the studied disease. We assumed that these factors may be cytokines: vascular endothelial growth factor (VEGF) and macrophage colony-stimulating factor (M-CSF).

VEGF has been described as an important regulator of angiogenesis, a crucial process of tumor invasion and progression [4]. Significantly increased levels of VEGF have been found in the serum or plasma of patients suffering from breast and gynecological tumors, for example, ovarian or cervical, as well as other kinds of cancers [5]. The* in vitro* and* in vivo* studies performed so far presented that the overexpression of this cytokine leads to cancer growth and metastasis, while the inhibition of VEGF resulted in the suppression of tumor development [6].

In contrast, M-CSF is a cytokine whose functions include regulation of hematopoietic lineages cells growth, proliferation, and differentiation [7]. M-CSF is produced pathologically by cancer cells. The overexpression of this cytokine has been detected in a variety of tumors, female reproductive tract cancers and breast, renal, colorectal, pancreatic, prostate, and head and neck tumors, and has been correlated with poor prognosis [8, 9]. What is interesting, circulating level of M-CSF has been found to be useful as a method of estimating patients' survival rates.

As VEGF and M-CSF play a significant role in carcinogenesis, the aim of the present study was to investigate the diagnostic power of the selected cytokines and a comparative marker CA 15-3 in breast tumor detection.

In this paper, the use of healthy volunteers and women with benign breast lesions together as a one control group better reflects the current population of women. The data obtained in this work may prove the usefulness of the analyzed parameters (separately and together) in the detection of BC, as a new diagnostic panel.

## 2. Materials and Methods

### 2.1. Patients

Table 1 shows the tested groups. The study included 120 breast cancer (BC) women diagnosed by the oncology group. The breast cancer patients were treated in the Department of Oncology, Medical University of Bialystok, Bialystok, Poland. Tumor classification and staging were done in accordance with the International Union Against Cancer Tumor-Node-Metastasis (UICC-TNM) classification. The breast cancer histopathology was established in all cases by tissue biopsy of mammary tumor or after surgery from tumor cancer tissues (all patients with* ductal adenocarcinoma*). The pretreatment staging procedures included physical and blood examinations, mammography, mammary ultrasound scanning, breast core biopsies, and chest X-rays.

In addition, radioisotopic bone scans, examination of bone marrow aspirates, and CT scans of brain and chest were performed when necessary. None of the patients had received chemotherapy or radiotherapy before blood sample collection.

The control group included 120 patients (60 patients with benign breast tumor,* adenoma, intraductal papilloma, fibroadenoma, mastopatia*, and 60 healthy untreated women) who underwent mammary gland examination performed by a gynecologist prior to blood sample collection. In addition, mammary ultrasound scanning was performed in all cases. The benign breast tumor histopathology was established in all cases by tissue biopsy of mammary tumor or after surgery.

The study was approved by the local Ethics Committee in Medical University of Bialystok (R-I-002/239/2014). All the patients gave their informed consent for the examination.

### 2.2. Biochemical Analyses

Venous blood samples were collected from each patient into a heparin sodium tube, centrifuged at 1000 rpm for 15 min to obtain plasma samples and stored at –85°C until assayed. The tested parameters were measured with the enzyme-linked immunosorbent assay (ELISA) (VEGF and M-SCF, Quantikine Human Immunoassay, R&D Systems Inc., Minneapolis, MN, USA) and chemiluminescent microparticle immunoassay (CMIA) (CA 15-3, Abbott, Chicago, IL, USA). According to the manufacturer's protocols, duplicate samples were assessed for each standard, control, and sample.

The intra-assay coefficient of variation (CV%) of CA 15-3 is reported to be 2.2% at a mean concentration of 27.0 U/mL (SD = 0.6). VEGF is reported to be 4.5% at a mean concentration of 235 pg/mL (SD = 10.6). M-CSF is reported to be 3.4% at a mean concentration of 227 pg/mL (SD = 7.7).

The interassay coefficient of variation (CV%) of CA 15-3 is reported to be 2.6% at a mean concentration of 27.0 U/mL (SD = 0.7). VEGF is reported to be 7.0% at a mean concentration of 250 pg/mL (SD = 17.4). M-CSF is reported to be 3.1% at a mean concentration of 232 pg/mL (SD = 7.3).

### 2.3. Statistical Analysis

In this analysis we have used healthy volunteers and women with benign breast lesions together as a one control group. This is in accordance with the latest published papers especially for ROC analysis [10–13]. Statistical analysis was performed by using STATISTICA 12.0. We have defined the receiver-operating characteristics (ROC) curve for all the tested parameters and CA 15-3. The construction of the ROC curves was performed using GraphROC program for Windows and the areas under ROC curve (AUCs) were calculated to evaluate the diagnostic accuracy and to compare AUCs for all tested parameters separately and in combination with a commonly used tumor marker (CA 15-3). Statistically significant differences were defined as comparisons resulting in *p* < 0.05.

The* cut-off* values were calculated by Youden's index (as a criterion for selecting the optimum* cut-off* point) and for each of the tested parameters they were as follows: VEGF, 70.25 pg/mL; M-CSF, 394.38 pg/mL; and CA 15-3, 18.30 U/mL.

## 3. Results 

Table 2 shows the sensitivity (SE) and specificity (SP) of the investigated parameters and CA 15-3. We indicated that the SE of the tested parameters in the total cancer group was the highest for CA 15-3 (83.75%) and slightly higher than that for VEGF (76.25%) and M-CSF (60%). Among all parameters, the highest SE in stage I of cancer was observed for VEGF (75%), in stage II of BC it was observed for VEGF and CA 15-3 (75%, equal for both parameters), and in stages III and IV of BC it was observed for CA 15-3 (95% and 100%, resp.).

The diagnostic SP of the tested parameters was the highest for M-CSF and VEGF (90% and 85%, resp.) and was higher than that for CA 15-3 (75%).

The combined analysis for VEGF or M-SCF with CA 15-3 in the total group of BC resulted in a high increase in SE in both cases (96.25% and 91.25%, resp.). A similar range in the total BC group was obtained for the combination of VEGF, M-SCF, and CA 15-3 (96.25%). In all combinations, SP dropped slightly in comparison to the analysis of single parameters.

The relationship between the diagnostic SE and SP is illustrated by the ROC curve. The area under the ROC curve (AUC) indicates the clinical usefulness of a tumor marker and its diagnostic power. It also quantifies the overall ability of the test to differentiate between the individuals with the disease and those without it. All data related to AUCs in different stages of BC (I–IV) are included in Table 3.

**Figure 1:**
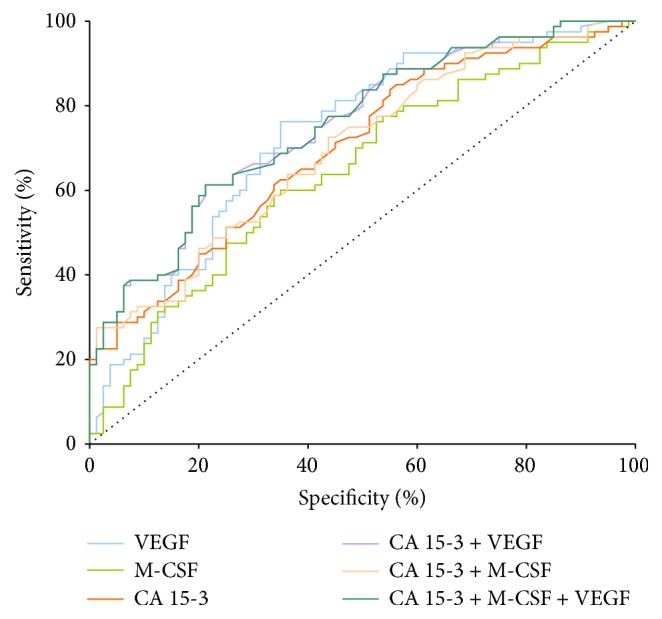
Diagnostic criteria of ROC curve for tested parameters and in combination with CA 15-3 in total BC group.

We noticed that the VEGF area under the ROC curve (0.729) in the total group of breast cancer was higher than the area of CA 15-3 (0.698) and M-CSF (0.645), statistically significantly larger in comparison to AUC = 0.5, borderline of the diagnostic usefulness of the test (*p* < 0.001 in all cases). The combined analysis of VEGF or M-SCF with CA 15-3 in the total group of BC resulted in a slight increase in AUCs in both cases (0.753 and 0.699, resp.), but a maximum range in the total BC group was obtained for the combination of VEGF, M-SCF, and CA 15-3 (0.754) (*p* < 0.001 in all cases) (Figure 1).

**Figure 2:**
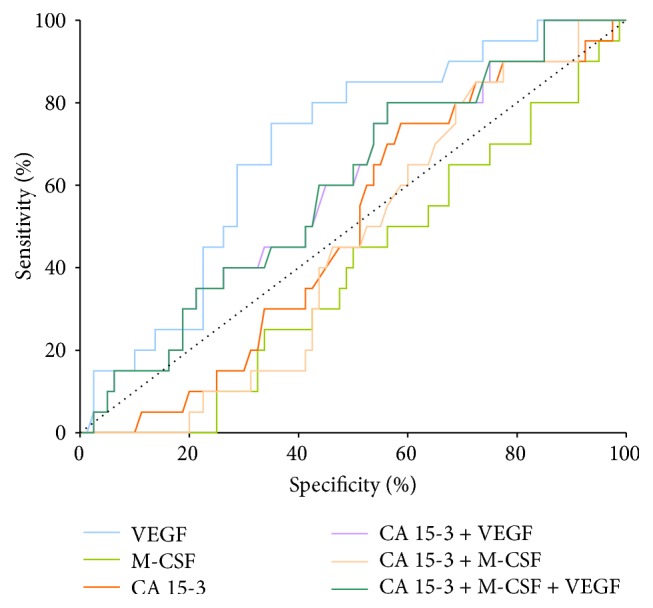
Diagnostic criteria of ROC curve for tested parameters and in combination with CA 15-3 in stage I of BC.

In stage I of BC the highest AUC of all the tested parameters was found in VEGF (0.691) and it was the only parameter which was statistically significantly larger in comparison to AUC = 0.5 (*p* < 0.002) (Figure 2).

**Figure 3:**
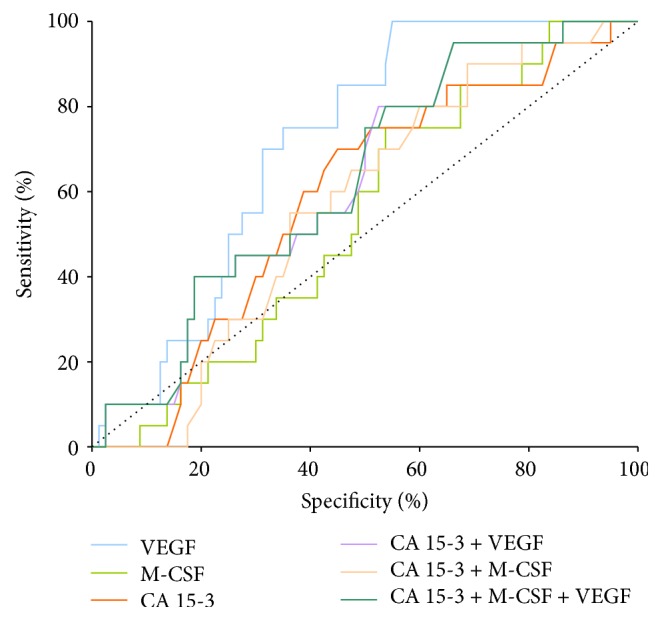
Diagnostic criteria of ROC curve for tested parameters and in combination with CA 15-3 in stage II of BC.

In stage II of BC the highest AUC of all tested parameters was also observed in VEGF (0.716; *p* < 0.001). The combined analysis of VEGF with CA 15-3 (0.629; *p* = 0.043) and combination of all tested parameters showed a slight decrease in AUC (0.629; *p* = 0.042) (Figure 3).

**Figure 4:**
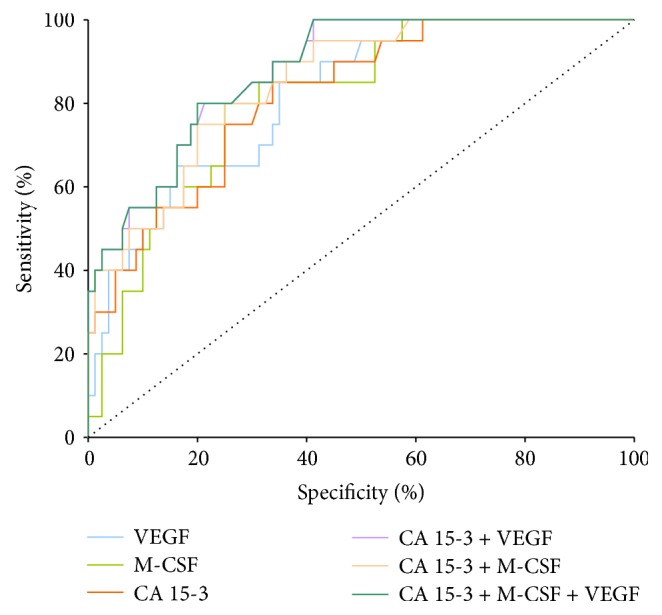
Diagnostic criteria of ROC curve for tested parameters and in combination with CA 15-3 in stage III of BC.

In stage III of BC the highest AUC of all the tested parameters was observed in CA 15-3 (0.819; *p* < 0.001) and it was slightly higher than VEGF (0.818; *p* < 0.001) and M-CSF (0.811; *p* < 0.001). The combined analysis of VEGF or M-CSF with CA 15-3 showed an increase in AUC values (0.878 and 0.850, resp.) (*p* < 0.001 in both cases), but the maximum range in stage III of BC was obtained for the combination of VEGF, M-SCF, and CA 15-3 (0.879; *p* < 0.001) (Figure 4).

**Figure 5:**
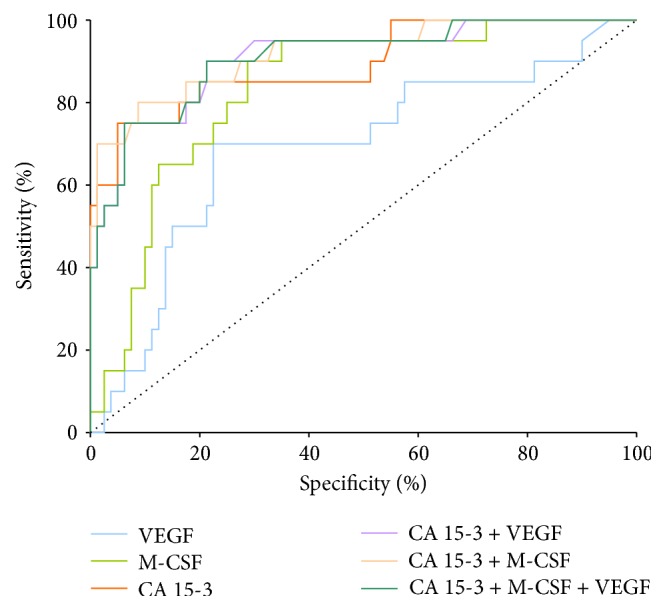
Diagnostic criteria of ROC curve for tested parameters and in combination with CA 15-3 in stage IV of BC.

In stage IV of BC the highest AUC of all the tested parameters was found in CA 15-3 (0.893; *p* < 0.001) and it was higher than M-CSF (0.834; *p* < 0.001) and VEGF (0.690; *p* = 0.008). The combined analysis of VEGF with CA 15-3 or all tested parameters showed an increase in AUC values (0.908; *p* < 0.001 in both cases), but the maximum range in stage IV of BC was obtained for the combination of M-SCF and CA 15-3 (0.921; *p* < 0.001) (Figure 5).

## 4. Discussion

Angiogenesis is a vital blood vessel formation process in tumor progression and nutrition. VEGF is considered to be an important factor in promoting angiogenesis and cell proliferation in many pathological conditions. High levels of VEGF have been found in different kinds of tumors, for example, gastric [14] or colorectal cancer [15], and also in gynecological malignancies such as ovarian [16] or cervical cancer [17]. High plasma levels of VEGF have been also found in breast cancer [5].

Tumor growth is influenced by a variety of external and internal factors. Our immune system (producing growth factors and cytokines) is one of the most important mediators involved in tumor development. M-CSF belongs to the group of hematopoietic growth factors (HGFs) which are overexpressed in many tumors. The main function of M-CSF is regulation and differentiation of hematopoietic progenitor cell growth. Its high levels have been found in gastric [18] and pancreatic cancer [19]. It has also been found in many types of gynecological malignancies, for example, ovarian [20, 21], cervical [22], or endometrial cancer [23], and it has also been found in breast cancer [8].

Sensitivity (SE) measures the proportion of positives that are correctly identified. In this study, the SE for CA 15-3 was the highest in the total group of breast cancer patients. However, in stages I and II of cancer it was the highest for VEGF which is much more important because such a high sensitivity (75%) allows us to confirm the occurrence of breast cancer in the earliest stages, while contributing to an increase in cancer detection, the course of which is often asymptomatic. Earlier diagnosis is associated with a greater chance of survival as well as quality and length of life of patients with BC. Similar data were observed in our previous studies [5, 8], where CA 15-3 had also the highest values in the total group, but what is more important is the fact that VEGF had a higher value in stage I of BC. However, in opposition to this paper, statistical analysis of those previous publications was conducted on groups of “breast cancer patients versus healthy women” only.

Other researchers, such as Motawa El Husseini et al. [24], have also indicated very high SE (83.93%) and SP (96.67%) for VEGF in BC diagnostics, but they conducted their study on 51 BC patients and only 30 healthy volunteers as a control group.

We have also observed similar data in other types of cancer, for example, in ovarian cancer [16]. Other researchers, for instance, Kozłowski et al. [25] in esophageal cancer (SE, 83%; SP, 70%) or Cao et al. [26] in lung cancer (SE, 81.8%; SP, 84.2%), have obtained similar results for VEGF.

The AUC represents the overall accuracy of a test, with a value approaching 1.0 indicating perfect SE and SP. According to this study, the ROC area of VEGF was the largest of all the tested parameters (even higher than CA 15-3 which is nowadays commonly used in the diagnosis of BC) and is the only parameter for which AUC was statistically significantly larger in comparison to AUC = 0.5 in all stages of BC (I–IV), not only in the total group. This is very important as it indicates higher usefulness of VEGF compared to CA 15-3 in the differentiation between BC and benign breast tumor.

Our results showed that the diagnostic power (AUC) of the tested parameters, especially VEGF, in the total group of BC patients was slightly lower than the one obtained by Zhang et al. [27] (0.788). The discrepancy between our research and that study may be related to a different number of patients involved in those studies. Other researchers such as Motawa El Husseini et al. [24] have obtained a higher AUC value for VEGF (0.938), but the control group in their study comprised only healthy women.

The diagnostic power of VEGF in the course of other tumors, for example, studies conducted in lung cancer by Cao et al. [26], revealed a slightly higher AUC value (0.855) than our outcome, which may be associated with different types of cancer. Other researchers, for example, Kozłowski et al. [25], have obtained slightly higher results for VEGF (0.865) in esophageal cancer. This may result from the fact that they conducted their study on 30 healthy volunteers in control group (without benign cancer patients). High importance of VEGF in those types of tumors points out that this cytokine seems to be a good biomarker for a variety of cancers, as shown by other researchers. In stage I of BC the highest AUC of all tested parameters was observed for VEGF. In our previous study in BC [28], which comprised BC patients and only healthy women as a control group, the highest AUC value was found for CA 15-3 (0.7068) and it was the only parameter for which AUC was statistically significantly larger in comparison to AUC = 0.5 (*p* = 0.002). Present statistical analysis with new, combined control group revealed even better results for tested cytokine (VEGF is a better marker than CA 15-3), which is additionally in opposition to the previous results obtained for M-CSF. In our other study [21] conducted in ovarian cancer, which compared M-CSF to HE4 and CA 125, the AUC value in stage I was 0.7676 (*p* < 0.001) and was significantly higher than that in this study.

In stage II of BC, only VEGF and the combined analysis of VEGF and CA 15-3 had a statistically significantly larger AUC in comparison to AUC = 0.5. In our previous study in BC [28] all the tested parameters had significant values (which might be related with the composition of the control group, only healthy subjects). In the study on ovarian cancer [21] the value of AUC for M-CSF was higher (similarly to stage I) than that in this study, but the control group in this study also comprised only healthy women.

In stages III and IV of BC, all the tested parameters had statistically significantly larger AUC in comparison to AUC = 0.5. In our previous study in BC [28] all the tested parameters also showed significant values similarly to the study conducted in ovarian cancer [21].

The combined analysis of VEGF or M-CSF with CA 15-3 resulted in an increase in SE and AUC values, which may be useful in the future diagnosis of this cancer. This study is also similar to our previous paper, indicating diagnostic usefulness of this biomarkers panel in cancer diagnostics. Better parameters were obtained in the combination of VEGF than M-CSF and CA 15-3. The combination of all three parameters did not affect the significant increase in SE, SP, or AUC, which may lead to the assumption that the combination of VEGF and CA 15-3 may be the best diagnostic panel in the diagnosis of BC.

## 5. Conclusions

In conclusion, our present results indicate the usefulness and a high diagnostic power of VEGF in the detection of breast cancer. Among the tested parameters, VEGF occurred to be the best candidate for cancer diagnostics (better than commonly used tumor marker, CA 15-3) especially in stages I and II of BC as well as in the differentiation between BC and benign breast tumor. M-CSF has shown low SE in stages I and II and was comparable to CA 15-3 and VEGF, SE, and AUC values in stages III and IV of BC. VEGF, especially in the combination with CA 15-3, showed the highest usefulness and diagnostic power in the detection of breast cancer and may indicate a new panel of biomarkers used in early diagnosis of BC.

## Figures and Tables

**Table 1:** Characteristics of breast cancer patients and control groups: benign breast tumor and healthy women.

Study group		Number of patients
Tested group	*Breast cancer patients*	120
	*Ductal adenocarcinoma*	120
	Median age (range)	54 (34–72)
	Tumor stage	
	I	29
	II	30
	III	31
	IV	30
	Menopausal status	
	(i) Premenopausal	51
	(ii) Postmenopausal	69

Control group	*Benign breast tumor patients*	60
	*Adenoma*	21
	*Intraductal papilloma*	18
	*Fibroadenoma*	11
	*Mastopatia*	10
	Median age (range)	44 (26–71)
	Menopausal status	
	(i) Premenopausal	29
	(ii) Postmenopausal	31
	*Healthy women*	60
	Median age (range)	48 (23–73)
	Menopausal status	
	(i) Premenopausal	26
	(ii) Postmenopausal	34

**Table 2:** Diagnostic criteria of tested parameters and in combined analysis with CA 15-3 in breast cancer patients.

Tested parameters	Diagnostic criteria (%)	Breast cancer				
		Total group	Stage I	Stage II	Stage III	Stage IV
VEGF	SE	76.25	75	75	85	70
	SP	85	85	85	85	85

M-CSF	SE	60	25	35	85	95
	SP	90	90	90	90	90

CA 15-3	SE	83.75	65	75	95	100
	SP	75	75	75	75	75

VEGF + CA 15-3	SE	96.25	90	95	100	100
	SP	65	65	65	65	65

M-CSF + CA 15-3	SE	91.25	80	85	100	100
	SP	67.5	67.5	67.5	67.5	67.5

VEGF + M-CSF + CA 15-3	SE	96.25	90	95	100	100
	SP	57.5	57.5	57.5	57.5	57.5

**Table 3:** Diagnostic criteria of ROC curve for tested parameters and CA 15-3.

Tested parameters	AUC	SE	95% C.I. (AUC)	*p* (AUC = 0.5)
*ROC criteria in breast cancer (total group)*				
VEGF	0.729	0.0400	0.650–0.807	**<0.001**
M-CSF	0.645	0.0436	0.559–0.730	**0.009**
CA 15-3	0.698	0.0410	0.618–0.779	**<0.001**
VEGF + CA 15-3	0.753	0.0377	0.679–0.826	**<0.001**
M-CSF + CA 15-3	0.699	0.0409	0.618–0.779	**<0.001**
VEGF + M-CSF + CA 15-3	0.754	0.0377	0.679–0.827	**<0.001**

*ROC criteria in breast cancer (stage I)*				
VEGF	0.691	0.0616	0.570–0.811	**0.002**
M-CSF	0.396	0.0655	0.267–0.524	1.889
CA 15-3	0.494	0.0647	0.367–0.621	1.073
VEGF + CA 15-3	0.595	0.0680	0.462–0.729	0.161
M-CSF + CA 15-3	0.455	0.0611	0.336–0.575	1.535
VEGF + M-CSF + CA 15-3	0.596	0.0679	0.463–0.729	0.1561

*ROC criteria in breast cancer (stage II)*				
VEGF	0.716	0.0524	0.613–0.818	**<0.001**
M-CSF	0.539	0.0639	0.414–0.664	0.544
CA 15-3	0.586	0.0665	0.456–0.716	0.196
VEGF + CA 15-3	0.629	0.0637	0.504–0.754	**0.043**
M-CSF + CA 15-3	0.568	0.0632	0.444–0.691	0.285
VEGF + M-CSF + CA 15-3	0.629	0.0637	0.505–0.754	**0.042**

*ROC criteria in breast cancer (stage III)*				
VEGF	0.818	0.0483	0.724–0.913	**<0.001**
M-CSF	0.811	0.0484	0.716–0.906	**<0.001**
CA 15-3	0.819	0.0490	0.723–0.915	**<0.001**
VEGF + CA 15-3	0.878	0.0376	0.804–0.952	**<0.001**
M-CSF + CA 15-3	0.850	0.0436	0.765–0.936	**<0.001**
VEGF + M-CSF + CA 15-3	0.879	0.0375	0.805–0.952	**<0.001**

*ROC criteria in breast cancer (stage IV)*				
VEGF	0.690	0.0717	0.549–0.831	**0.008**
M-CSF	0.834	0.0461	0.744–0.925	**<0.001**
CA 15-3	0.893	0.0450	0.805–0.982	**<0.001**
VEGF + CA 15-3	0.908	0.0390	0.832–0.985	**<0.001**
M-CSF + CA 15-3	0.921	0.0368	0.848–0.993	**<0.001**
VEGF + M-CSF + CA 15-3	0.908	0.0387	0.832–0.984	**<0.001**

*p*, statistically significantly larger AUCs compared to AUC = 0.5.
